# Flow synthesis of a versatile fructosamine mimic and quenching studies of a fructose transport probe

**DOI:** 10.3762/bjoc.9.238

**Published:** 2013-10-07

**Authors:** Matthew B Plutschack, D Tyler McQuade, Giulio Valenti, Peter H Seeberger

**Affiliations:** 1 Department of Chemistry and Biochemistry, Florida State University, Tallahassee, FL 32306, USA; 2 Max Planck Institute of Colloids and Interfaces, Am Mühlenberg 1, 14476 Potsdam, Germany

**Keywords:** flow chemistry, fructose mimic, gluts, NBDM

## Abstract

We describe the synthesis of 1-amino-2,5-anhydro-D-mannose (“mannitolamine”), a key intermediate to the 7-nitro-1,2,3-benzadiazole conjugate (NBDM), using commercially available fluidic devices to increase the throughput. The approach is the first example of a flow-based Tiffeneau–Demjanov rearrangement. Performing this step in flow enables a ~64-fold throughput enhancement relative to batch. The flow process enables the synthesis to be accomplished three times faster than the comparable batch route. The high throughput enabled the production of larger quantities of the fluorescent fructose transport probe NBDM, enabling us to measure key photophysical properties that will facilitate future uptake studies.

## Introduction

The impact of dietary fructose on human health is not well-understood. A growing body of work suggests that those eating diets high in fructose exhibit increased rates of metabolic disorders and aggressive cancers [1–2]. Since the landmark reports from Warburg [3], researchers have recognized that cancer cells/tissues consume more carbohydrates than normal tissues, fueling rapid growth and proliferation [4]. While cancer cells utilize multiple strategies to increase carbohydrate consumption, one mechanism is to increase passive glucose transporter (Glut) expression [2,5]. Uncharacteristic Glut expression is now implicated as a hallmark of not just cancer but also metabolic disorders [1]. Abnormal Glut expression is observed in the pancreatic islets and hepatic cells of people with diabetes, which may explain glucose insensitivity and the progression of non-alcoholic fatty liver disease [6–7].

Passive carbohydrate transporters are well-known targets for carbohydrate-based probes [8]. The positron emission tomography (PET) tracer 2-deoxy-2-[^18^F]fluoro-D-glucose (FDG) primarily targets Glut1 [9]. Although FDG is an effective tumour probe [10], Glut1 is expressed in every type of tissue and this prevalence often results in false positive tests [11–12]. Unlike Glut1, the fructose-specific transporter Glut5 is expressed in fewer tissues [2]. We are developing probes that are selectively transported by Glut5. Using design principles gleaned from the Holman group [13–15] as well as other fructose analogue research [16], we synthesized NBDM [17] and demonstrated that this probe is transported into cancer cells known to overexpress Glut5 and poorly transported into cells known to express little Glut5 [2,5,18]. We demonstrated that the transport is inhibited by fructose but not by other dietary sugars (glucose, glucosamine) [17]. Furthering our understanding of fluorescent probes like NBDM will expedite the development of Glut5-specific PET compounds which could be non-invasive tools for determining Glut5 expression in vivo and provide a means for monitoring the onset and progression of metabolic syndrome and aggressive cancers.

The promising initial results obtained with NBDM prompted us to synthesize larger quantities of material. More NBDM is required to examine uptake across many cell lines and with access to amine **3**, we can prepare analogues with different fluorophores or other types of tags. Finally, access to more NBDM will enable assessment of probe photophysical properties as a function of concentration and in the presence of potential quenchers. This increased understanding will be critical when probing uptake into various biological systems where cell staining techniques or supplemental amino acids are used. Herein, we report an efficient flow synthesis of amine **3** that enabled an increase in scale as well as a reduction in the time needed to prepare NBDM. We also present fluorescent properties of NBDM under conditions relevant to future cellular studies.

## Results and Discussion

**Scheme 1:**
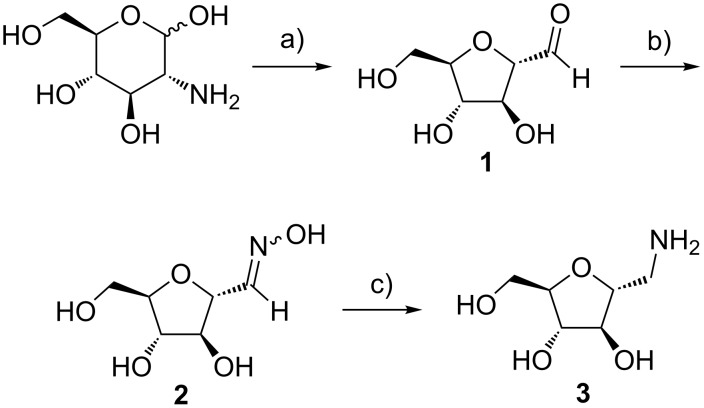
Batch synthesis of NBDM. a) NaNO_2_, Amberlite IR-120 H^+^ (100 mL), 0 °C, water, 4 h. b) NH_2_OH·HCl, NaOAc, MeOH, rt, 6 h. c) H_2_, Pd/C (10%), 4.4% formic acid, MeOH, rt, 10 h.

**Scheme 2:**
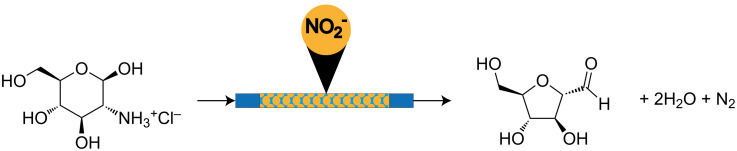
The initial polymer-supported nitrite set up. A solution of glucosamine hydrochloride was passed over the resin into a round bottom flask and then stirred with heating.

**Synthesis:** The batch synthesis of 1-amino-2,5-anhydro-D-mannitol was reported by Claustre et al (Scheme 1). We used their basic approach, but improved throughput significantly [19]. The synthesis began with a Tiffeneau–Demjanov rearrangement of glucosamine·HCl using an acidic resin and NaNO_2_ to make nitrous acid in situ (Scheme 2). The original conditions required neutralization by a basic resin. After rinsing both resins, a dilute aqueous solution of **1** resulted and overnight lyophilisation was required to isolate the product. Because the conditions are not easily integrated into a continuous process, we sought alternative approaches.

Our initial approach was to continue using a resin (Amberlite IRA-900) supporting nitrite in flow. While the use of supported reagents in flow is now well-established, the use of supported nitrite has not been widely adopted [20]. Amberlite IRA-900 was exchanged by eluting the chloride ion from the resin using a 1 M solution of sodium nitrite until no more chloride was observed via an AgNO_3_ test. Once the exchange was complete, the column was washed with deionized water. The column was first assessed by flowing a 0.2 M aqueous solution of D-(+)-glucosamine·HCl into a round bottom flask. Our initial experiment was performed at room temperature and took two days to reach completion. Heating the reaction to 40, 60 or 70 °C resulted in a clean acceleration in reaction rate.

**Scheme 3:**
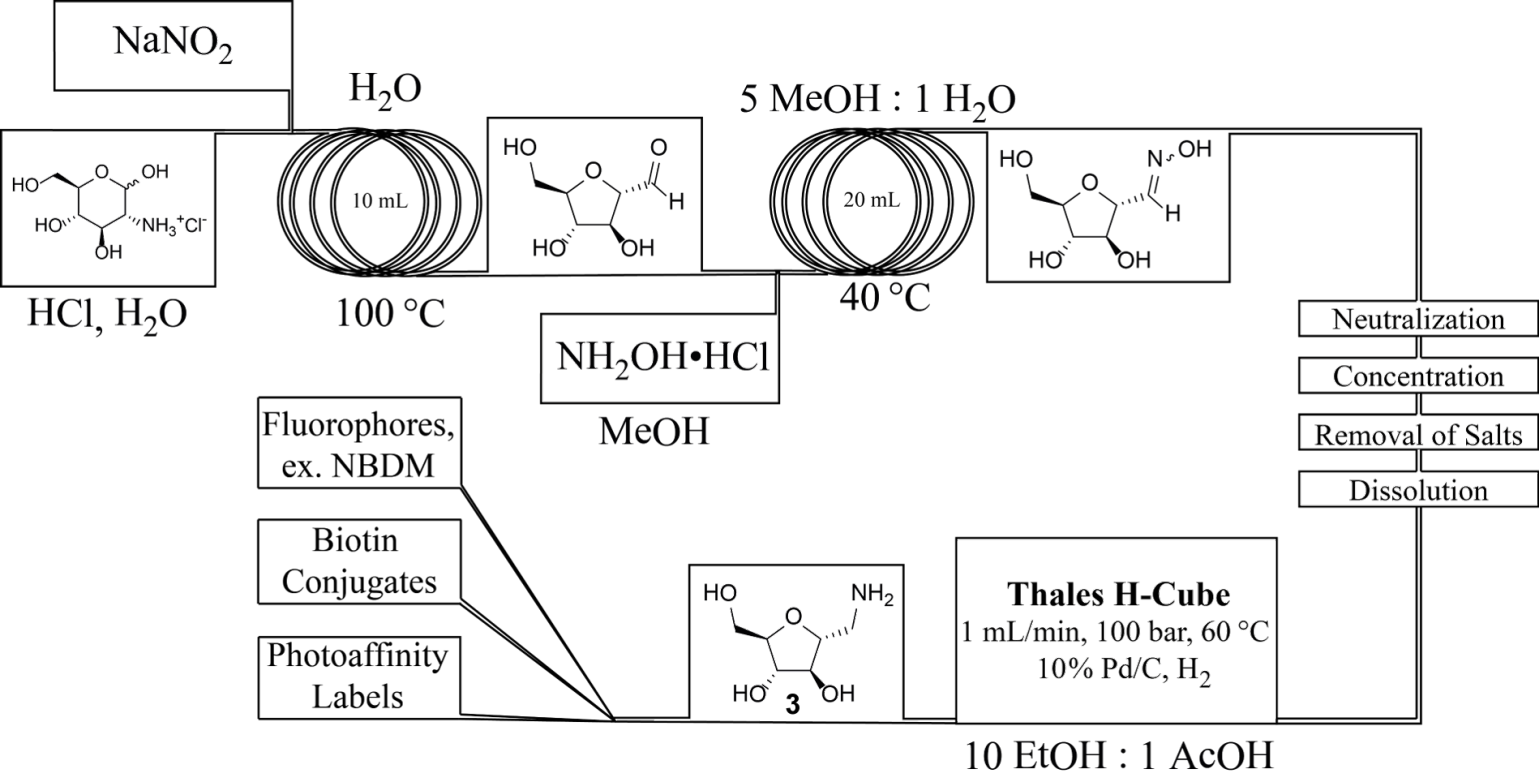
Continuous flow synthesis of the key intermediate 1-amino-2,5-anhydro-D-mannose (**3**).

Although the supported nitrite approach at elevated temperatures provided high yields of the desired product, the process was slow and throughput was restricted by the resin loading. In addition, attempts to increase concentration or use organic co-solvents further reduced the efficiency. Based on these problems, we turned our attention to Tiffeneau–Demjanov conditions using a catalytic amount of acid and sodium nitrite in water. Flow conditions enabled the use of high concentrations (1.0 M) and temperatures (100 °C) even though the reaction evolves large quantities of nitrogen gas. At a flow rate of 5 mL/min at 100 °C using a 10 mL reactor (2 min residence time), we achieved a throughput of 800 mg/min (Scheme 3). Table 1 shows a comparison between previously reported batch conditions and our continuous flow conditions and illustrates that this first step has a throughput more than 63-fold higher than the batch Tiffeneau–Demjanov conditions.

**Table 1:** Batch versus flow comparison.

Time (h)	Compound	Batch yield (g)	Flow yield^a^ (g)

4	**1**	3.06	194.4
6	**2**	2.63	63.8
10	**3**	2.21	4.8

^a^Flow yield is based off of throughput for the equivalent amount of time the batch conditions required. Throughput was estimated by the quantity of starting material pumped through the reactor. No percent yields were obtained because the crude mixtures could be used in the subsequent steps.

The concentrated Tiffeneau–Demjanov reaction not only increased the throughput but also enabled oxime formation without the removal of water. Using excess hydroxylamine hydrochloride (4 equiv) in methanol resulted in full conversion to compound **2**. At a total flow rate of 5 mL/min at 60 °C using a 20 mL reactor (4 minutes residence time), we achieved a throughput of 177 mg/min (24-fold improvement relative to batch).

The hydrogenolysis of the oxime illustrates a flow chemistry challenge. The output from the oxime step contains excess hydroxylamine and salts carried from the first step that poisoned the packed-bed catalyst we screened. In theory, continuous purification could remove these materials but existing strategies do not enable removal of water soluble byproducts from a water soluble product. Thus, we obtained crude **2** by simple work up: neutralization, concentration, precipitation of salts using tetrahydrofuran, filtration, concentration and dissolution in 10:1 ethanol/acetic acid. This solution was converted to amine **3** using an H-Cube (commercially available from Thales). With a flow rate of 1 mL/min at 100 bar and 70 °C using a 10% Pd/C CatCart, a throughput of 8 mg/min was achieved. The crude reaction solution was pure by NMR and was used without further purification. As shown in Table 1, the hydrogenolysis represents a bottleneck in the synthesis of compound **3** because the throughput drops down to only 2-fold enhancement relative to batch. We predict that this throughput could be significantly improved if the wider range of catalysts were screened.

The fructose analog probe NBDM was then produced by combining the concentrated output from the H-Cube in saturated sodium bicarbonate (0.4 M) with a 0.4 M solution of NBD-Cl. This step can be conducted in flow as well as in batch with no significant difference in yield or productivity. The low yield (20–30%) of NBDM may be the result of competitive reactivity between the NBD-Cl and the hydroxy groups present on **3**. When 1 M solutions of sodium bicarbonate are used instead of saturated sodium bicarbonate, the resulting TLCs indicate the formation of more byproducts and the lower isolated yields of NBDM (<20%) also support this hypothesis. We are confident that a completely continuous high-throughput process to **3** could be realized with improvements in continuous extraction techniques. That being said, the semi-continuous approach we have defined here results in significant improvements compared to the original batch conditions.

Many fluorophores and biologically relevant tags have been developed for conjugation to amines. For this reason, amine **3** was of particular interest. Likely, **3** will be a key branch-point for the synthesis of numerous biologically active conjugates and our improved production of **3** will provide significantly greater quantities of conjugates. In particular, we can now produce NBDM using this system in a single day which is a 3-fold improvement relative to the original process. This rapid access to more material enabled us to begin to assess the fluorescent properties as a function of typical quenchers used in cell-staining as well as intrinsic quenchers found in cells. Without easy access to NBDM, the use of this compound in biological experiments would supersede the investigation of its fluorescent properties.

**Fluorescence**: Fluorescence in biological systems is often complicated by fluorophore quenching. Alexa fluorophores can be quenched by certain amino acids and NBD is known to self-quench at high concentrations [21–22]. Trypan blue is routinely used to quench autofluorescence in confocal fluorescence microscopy and fluorescence activated cell sorting (FACS) [23–24], and dyes like Bromophenol Blue, Brilliant Blue R, and Methylene Blue have been applied to colorimetric cytotoxicity assays as well as in vitro staining [25–28].

**Figure 1:**
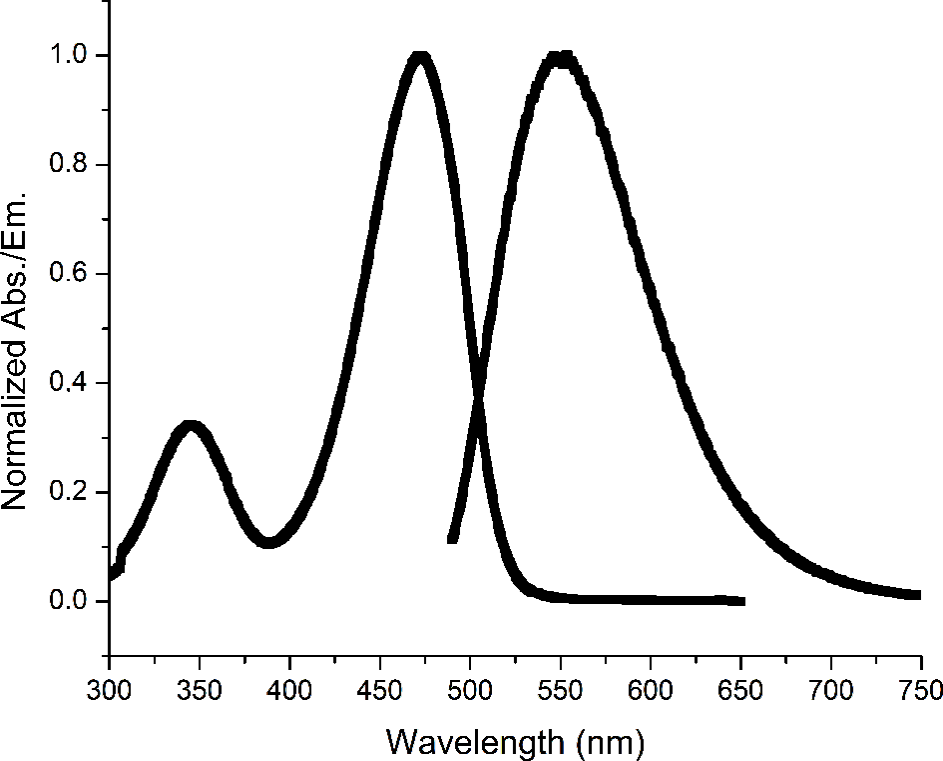
Normalized NBDM absorption and emission, 40 µM and 2 µM.

To better understand the behaviour of NBDM, we measured fluorescence at various concentrations and in the presence of various dyes, amino acids and sugars. The absorption and emission spectra are shown in Figure 1. The quenching experiments were carried out by measuring emission intensity at 546 nm (ex. 472 nm) as a function of NBDM concentration or quencher concentration. For each quenching experiment, 3–6 replicate fluorescence measurements were taken using a 96-well plate and a plate reader.

**Figure 2:**
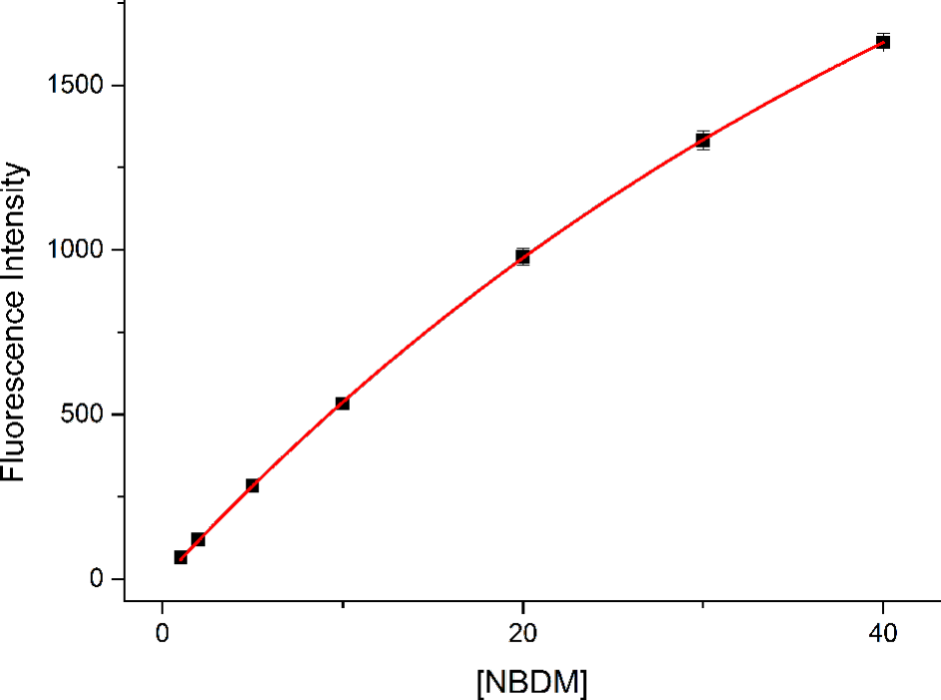
NBDM fluorescence from 1–40 µM (PBS buffer). The data set was plotted in OriginPro 8.6 and fitted using a self-quenching model, 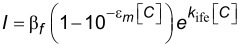 with direct weighting.

In our initial publication describing the uptake of NBDM into MCF-7 cells, we measured uptake as a function of concentration over a range of 1–40 μM (Figure 2) [17]. While we did not expect to observe significant self-quenching over this range, we measured the fluorescent intensity of NBDM from 1–40 μM (1X phosphate buffer solution). As expected, NBDM does not exhibit significant self-quenching over this range. The slight curvature that exists suggests that only modest self-quenching occurs (See Supporting Information File 1 for fitting data).

**Figure 3:**
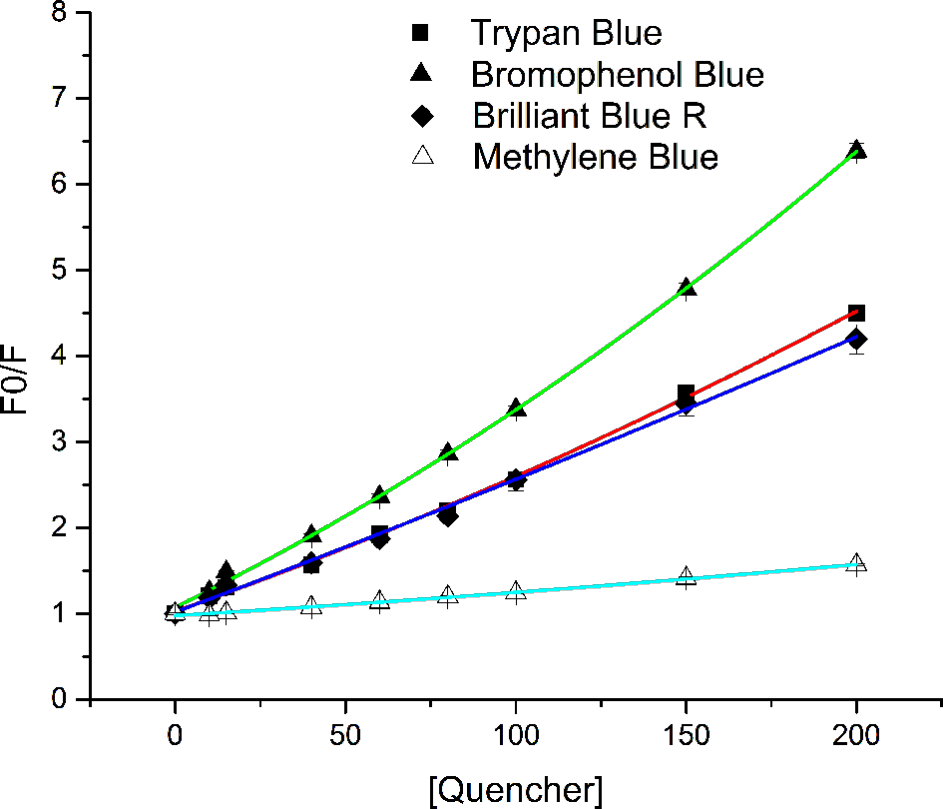
Comparison of quenching 2 µM NBDM, as measured by fluorescence intensity of Trypan Blue, Bromophenol Blue, Brilliant Blue R, and Methylene Blue. Each data set was plotted in OriginPro 8.6 and fitted using a polynomial fit (order = 2) with direct weighting.

Often multiple dyes are used to locate or identify cells by selective staining. The fluorescence data for NBDM in the presence of four commonly used dyes is shown in Figure 3. These data do not fit a simple Stern–Volmer relationship (see Supporting Information File 1), which indicates that dynamic quenching is not the only mechanism. The data do, however, fit well to a composite Stern–Volmer model that accounts for both dynamic and static quenching.

**Figure 4:**
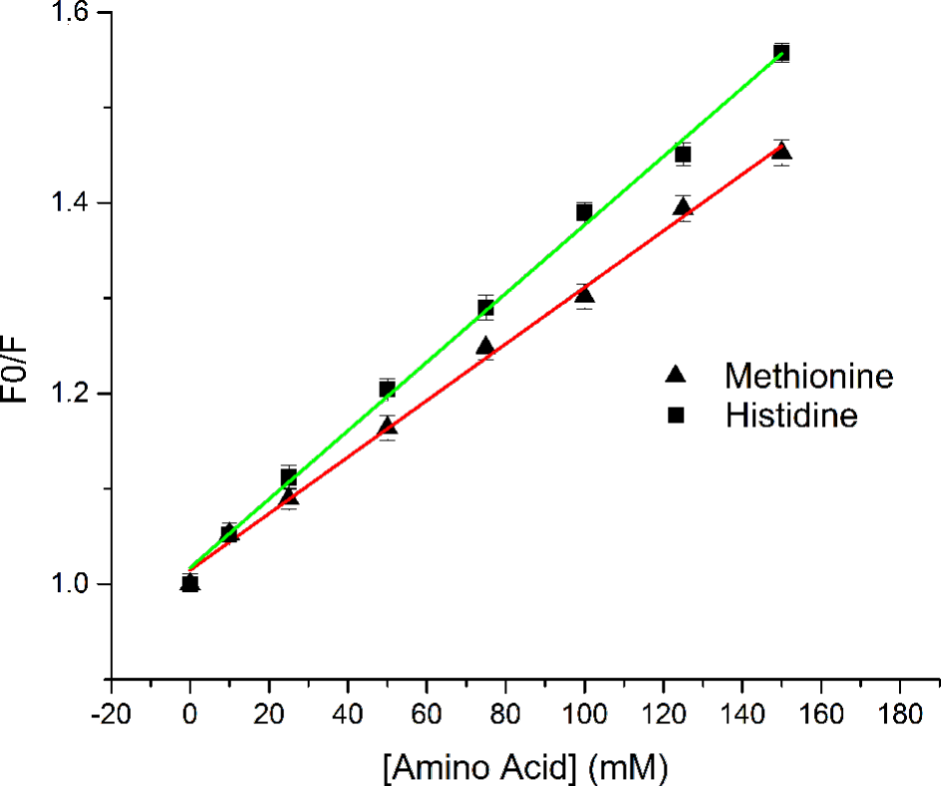
Comparison of quenching 2 µM NBDM, as measured by fluorescence intensity of methionine and histidine. Each data set was plotted in OriginPro 8.6 and fitted using a linear fit with direct weighting.

Free amino acids at high concentrations can also quench fluorophores. To access the propensity for NBDM to be quenched by amino acids, we measured fluorescence in the presence of varying concentrations of alanine, glutamine, lysine, tyrosine, methionine and histidine. As expected, the amino acids lacking functionality known to quench fluorophores (alanine, glutamine and lysine) did not quench NBDM at concentrations as high as 50 mM. Interestingly, tyrosine did not quench NBDM fluorescence even at concentrations as high as 2 mM (solubility limit for tyrosine). Methionine and histidine, however, did quench NBDM via a dynamic mechanism (Figure 4).

Carbohydrate–carbohydrate and carbohydrate–aromatic ring interactions are well-known [29–30]. Based on these interactions, we hypothesized that abnormal fluorescence behavior may be exhibited at high carbohydrate concentrations. This issue is significant because sugar uptake studies are often performed in the presences of added non-labeled sugars such as glucose, fructose or glucosamine. To assess our hypothesis, we measured the fluorescence of NBDM in the presence of glucose, fructose and glucosamine. No NBDM quenching was observed even at sugar concentrations as high as 100 mM.

## Conclusion

In conclusion, we report the flow synthesis of the fluorescent fructose mimic NBDM. While we demonstrated for the first time that resin-supported nitrite ions can facilitate Tiffeneau–Demjanov rearrangements, we found that solution phase rearrangements were superior resulting in throughput gains >63-fold relative to batch conditions. We also demonstrated that the output of the Tiffeneau–Demjanov rearrangement reactor could be telescoped into the oxime reactions. The oxime reaction was very efficient exhibiting throughput gains as high as 24-fold over batch. In addition, we identified extraction and hydrogenation bottlenecks. Despite these limitations, the synthesis of NBDM can now be achieved in one day as opposed to three days in batch. The access to larger quantities to NBDM enabled us to assess the probe’s quenching properties as a function of concentration and in the presence of various quenchers. These data are critical for future uptake studies that use confocal fluorescence microscopy or FACS strategies.

## Supporting Information

File 1 Experimental part.
