# Supplementary material for: Flow synthesis of a versatile fructosamine mimic and quenching studies of a fructose transport probe
Source: Beilstein J Org Chem. 2013 Oct 7;9:2022–7. doi: 10.3762/bjoc.9.238 (PMC3817519; doi:10.3762/bjoc.9.238)
Supplement: File 1 — Experimental part. [file Beilstein_J_Org_Chem-09-2022-s001.pdf]

Supporting Information

for

Flow synthesis of a versatile fructosamine mimic and quenching studies of a fructose transport probe

Matthew B. Plutschack[1,2], D. Tyler McQuade*[1,2], Giulio Valenti[2] and Peter H. Seeberger[2]

Address: [1]Department of Chemistry and Biochemistry, Florida State University, Tallahassee, FL 32306, USA and [2]Max Planck Institute of Colloids and Interfaces, Am Mühlenberg 1, 14476 Potsdam, Germany

Email: D. Tyler McQuade **-** **mcquade@chem.fsu.edu**

*Corresponding author

Additional material

1. Reagents and instrumentation

D-(+)-Glucosamine hydrochloride and 4-chloro-7-nitrobenzofurazan (NBDCl) were purchased from Alfa Aesar. The Bromophenol Blue and 10% Pd/C was purchased from Acros. The 10% Pd/C was packed into CatCarts from Thales. The Brilliant Blue R and glucose were purchased from Sigma Aldrich. The Methylene Blue is from Fischer Scientific and the Trypan Blue is from Carl Roth. Sodium nitrite and sodium bicarbonate were purchased from Grüssing GmbH. The amino acids were purchased from Degussa-Hüls AG. The Vapourtec R-2+ model and Thales H-Cube were used for the continuous flow synthesis of NBDM. ^{1}H NMR spectra were obtained using a Varian 400 MHz MR System and are reported in parts per million (ppm) relative to the solvent resonances (δ), with coupling constants (J) in Hertz (Hz). UV–vis spectra were recorded on a Shimadzu UV-Mini-1240 UV–vis Spectrophotometer. Fluorescence intensity measurements were taken using a Molecular Devices Spectra Max M5 plate reader. Column chromatography was performed using Fluka technical grade silica gel (230–400 mesh). Purification of NBDM was performed using a Biotage flash chromatography system equipped with fraction collector.

2. 2,5-Anhydro-D-mannose (1)

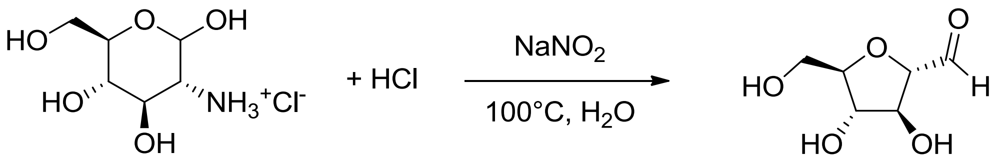

1

A 1.33 M solution of D-(+)-glucosamine HCl and a 6 M solution of $NaNO_2$ was reacted in the Vapourtec at flow rates of 4 mL/min and 1 mL/min respectively. The 10 mL reactor was heated at 100 °C. A 25 bar backpressure regulator was used to maintain a constant pressure and flow rate (Figure S2). TLC (4:1 ethyl acetate/methanol) confirmed full conversion to the product (R_f = 0.40). The crude ^{1}H NMR of 30 µL of crude reaction mixture added to 300 µL of D_2O, also confirmed full conversion.

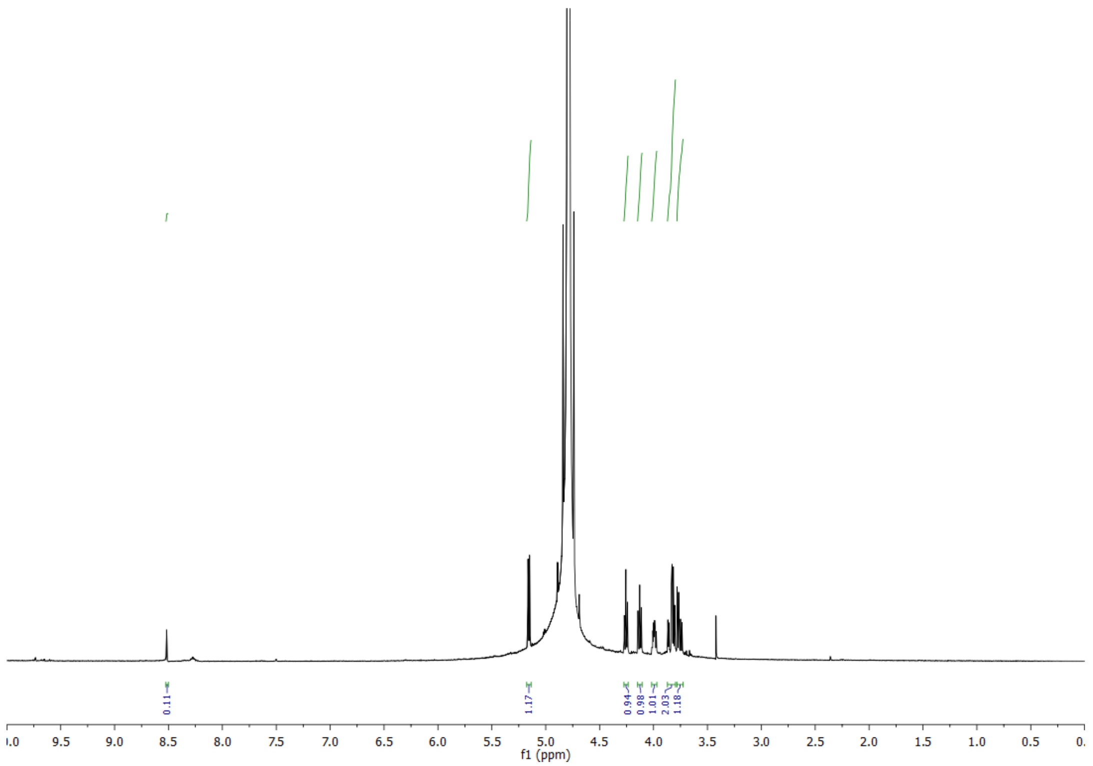

Figure S1: Crude ^{1}H NMR of **1** reaction output, 30 µL of crude reaction mixture added to 300 µL of D_2O. The spectrum is consistent with reported literature.

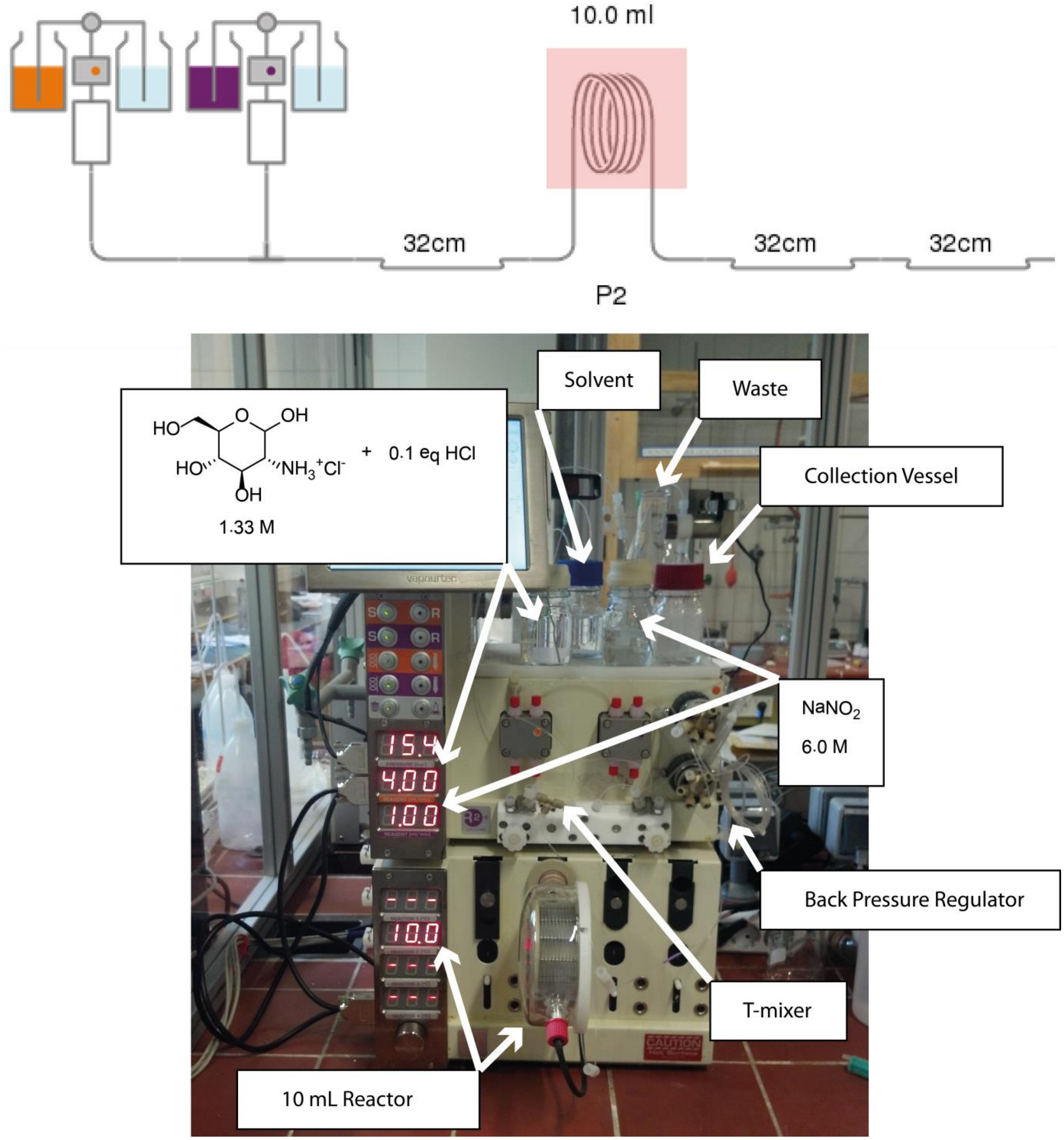

Figure S2: Experimental setup for of **1** using the Vapourtec R-2+ device.

3. 2,5-Anhydro-D-mannose oxime (2)

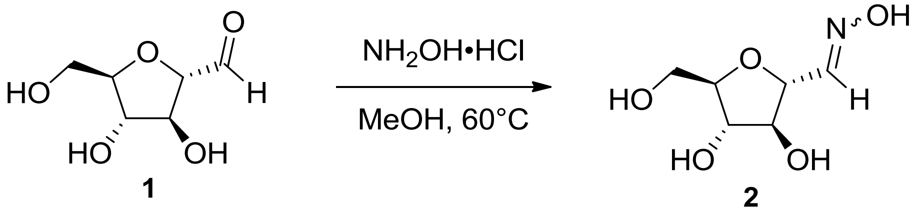

1 **2**

The 1.0 M solution of reaction ouput of **1** and a 1.0 M solution of NH_2OH HCl in methanol was reacted in the Vapourtec at flow rates of 1 mL/min and 4 mL/min respectively. The two 10 mL reactors were heated at 60 °C. A 25 bar backpressure regulator was used to maintain a constant pressure and flow rate (Figure S4). TLC (4:1 ethyl acetate/methanol) confirmed full conversion to the oxime (R_f = 0.69). The resulting solution was neutralized with sodium bicarbonate and stirring at room temperature. The solids were filtered and the supernatant was concentrated to a oily solid. This was taken up in THF, filtered through activated carbon and concentrated to a yellow oil. Crude ^{1}H NMR of the oil in methanol-d_4, also confirmed full conversion. This oil was dissolved and used without further purification.

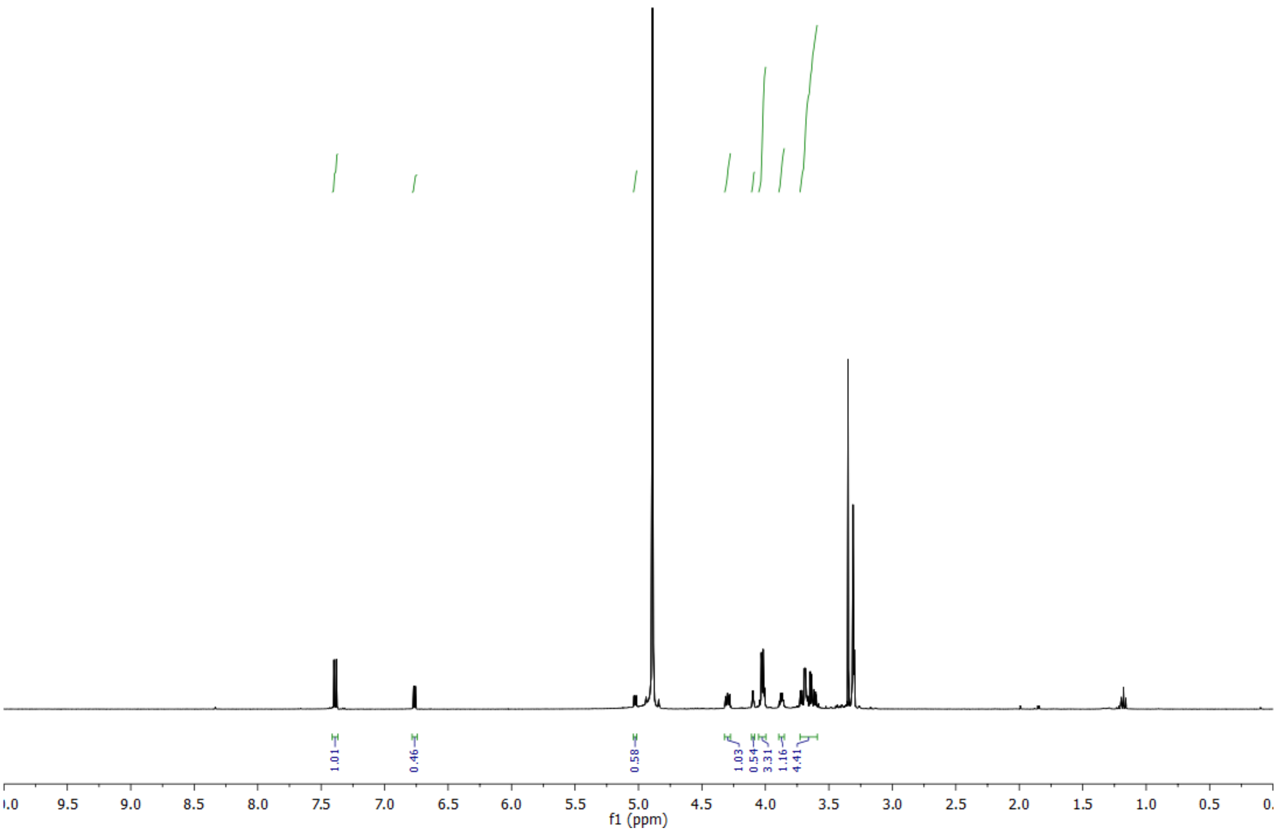

Figure S3: ^{1}H NMR of crude **2** in methanol-d_4 after work up. Spectrum is consistent with reported literature.

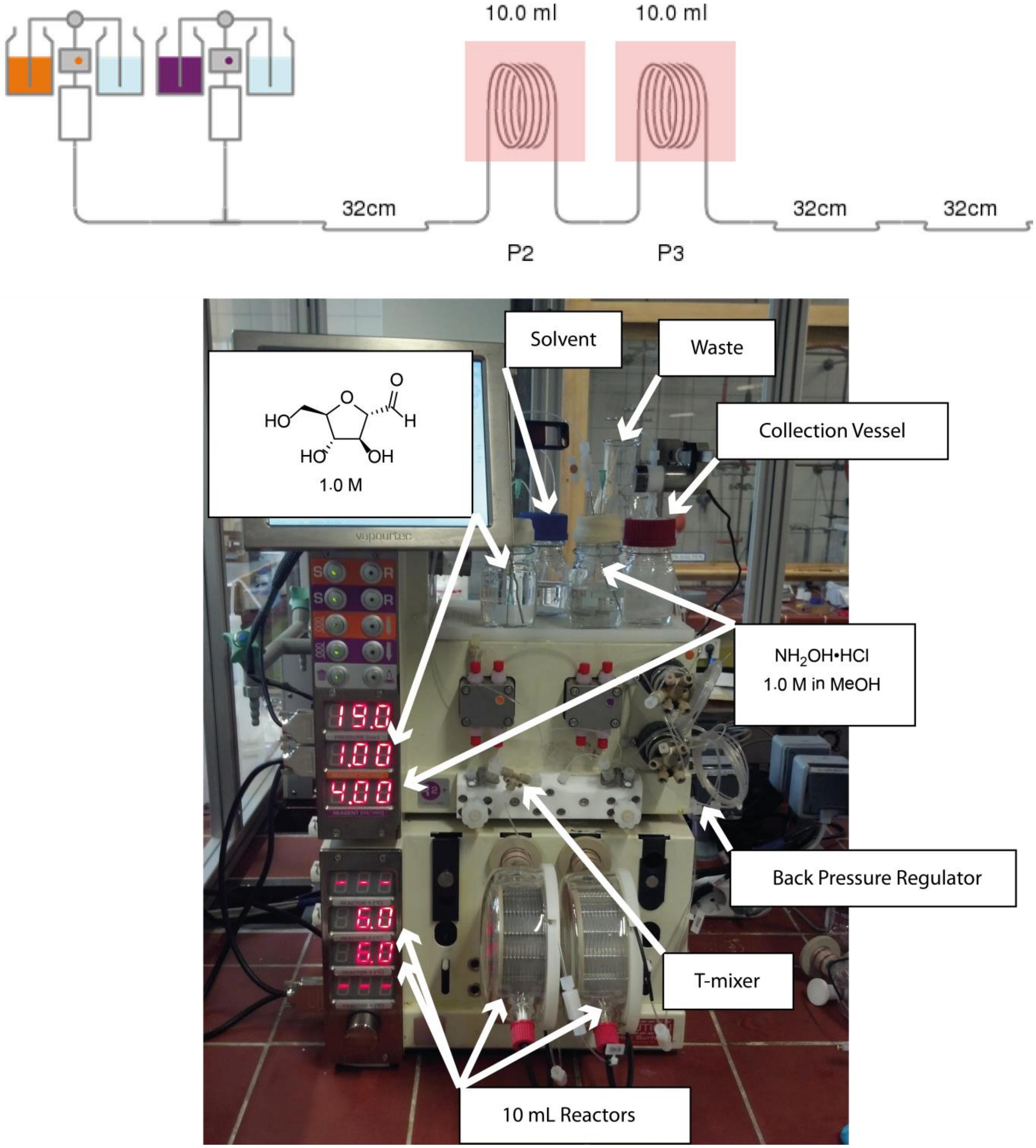

Figure S4: Experimental setup for **2** using the Vapourtec R-2+ device.

4. 1-Amino-2,5-anhydro-D-mannitol (3)

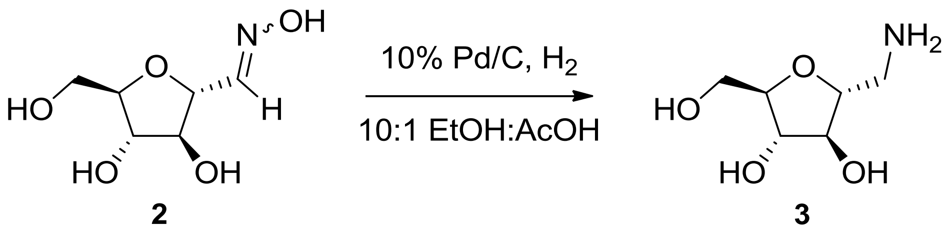

A 0.05 M solution of crude **2** in 10:1 ethanol/acetic acid was reacted in the Thales H-cube using a 10% Pd/C catalyst at flow rate of 1 mL/min at 70 °C and 100 bar (Figure S9). Concentrations of the collected solution resulted in a clear oil which was used directly in the next step.

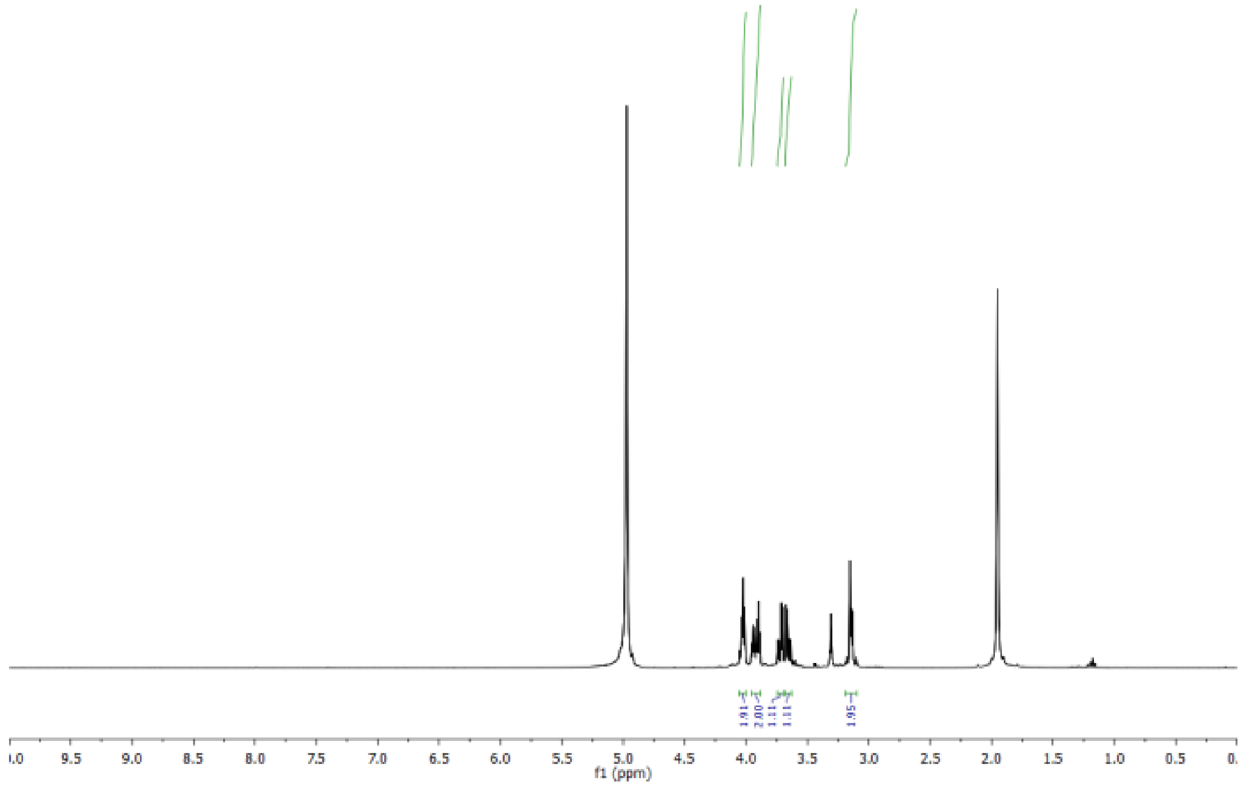

Figure S5: ^{1}H NMR of crude **3** in methanol-d_4. Spectrum is consistent with reported literature.

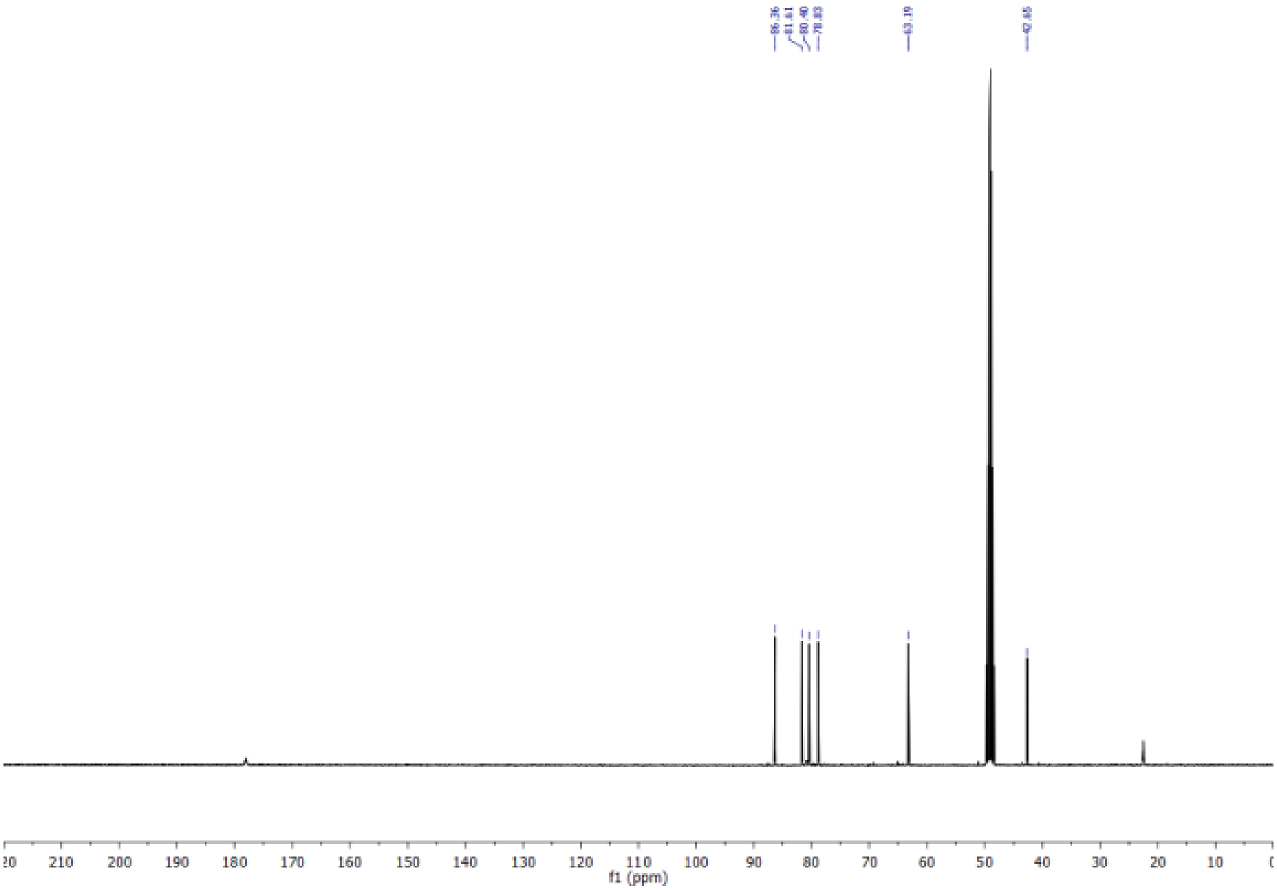

Figure S6: ^{13}C NMR of crude **3** in methanol-d_4. Spectrum is consistent with reported literature.

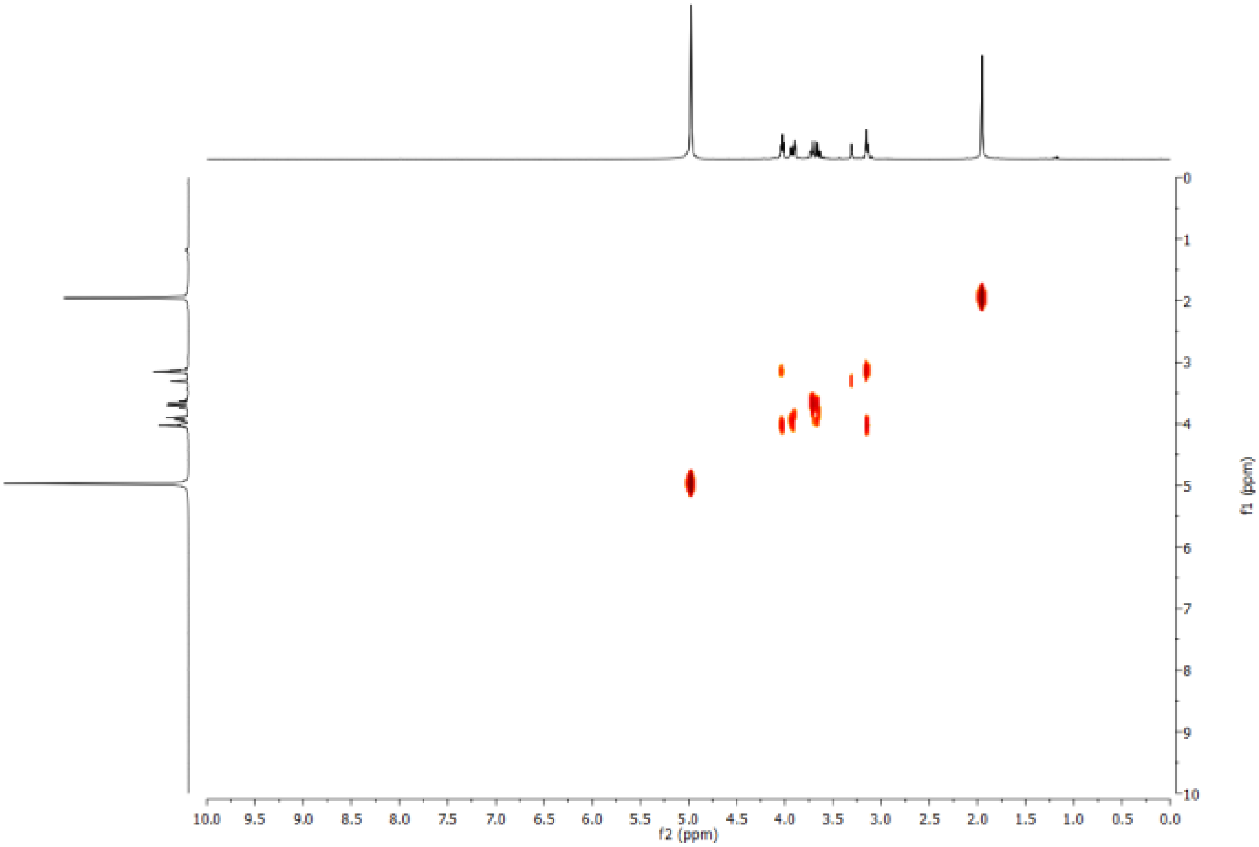

Figure S7: COSY of crude **3** in methanol-d_4.

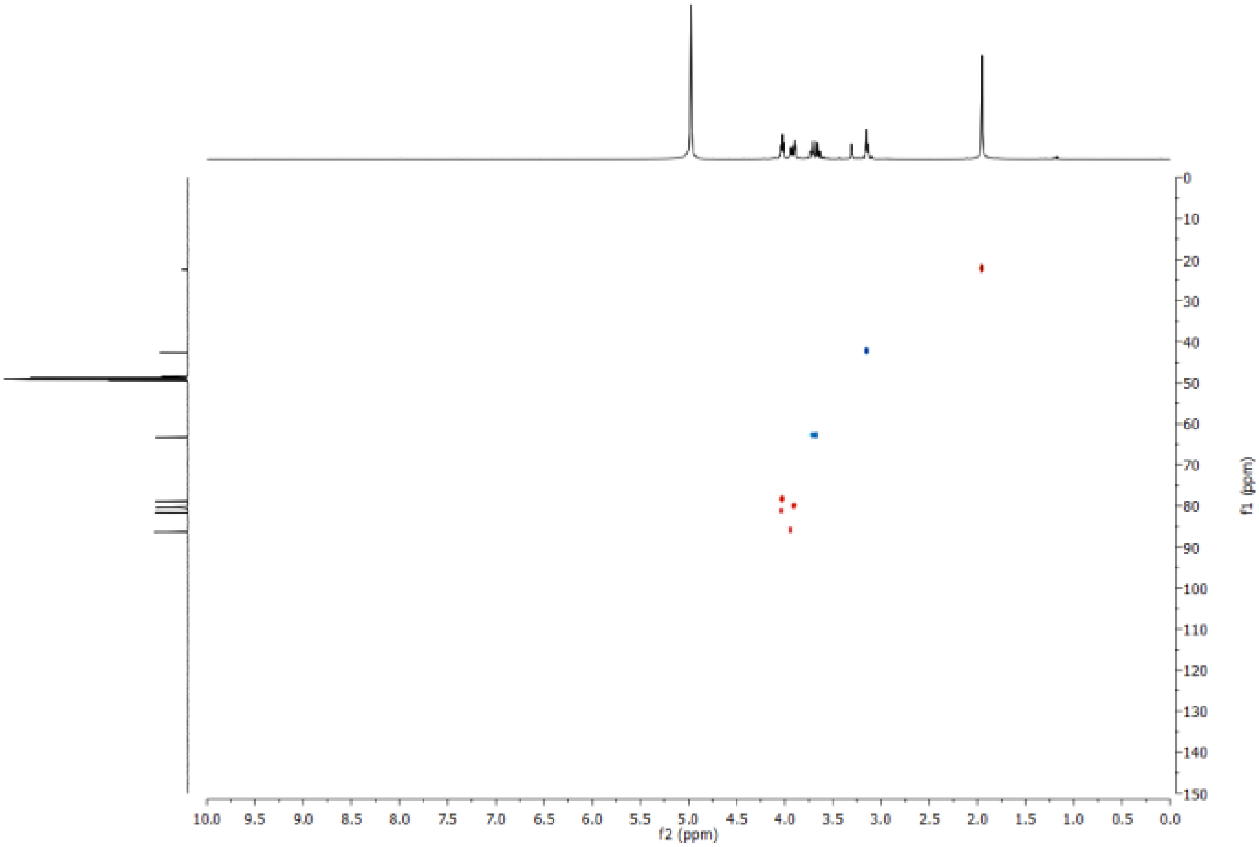

Figure S8: HSQC of crude oil of **3**.

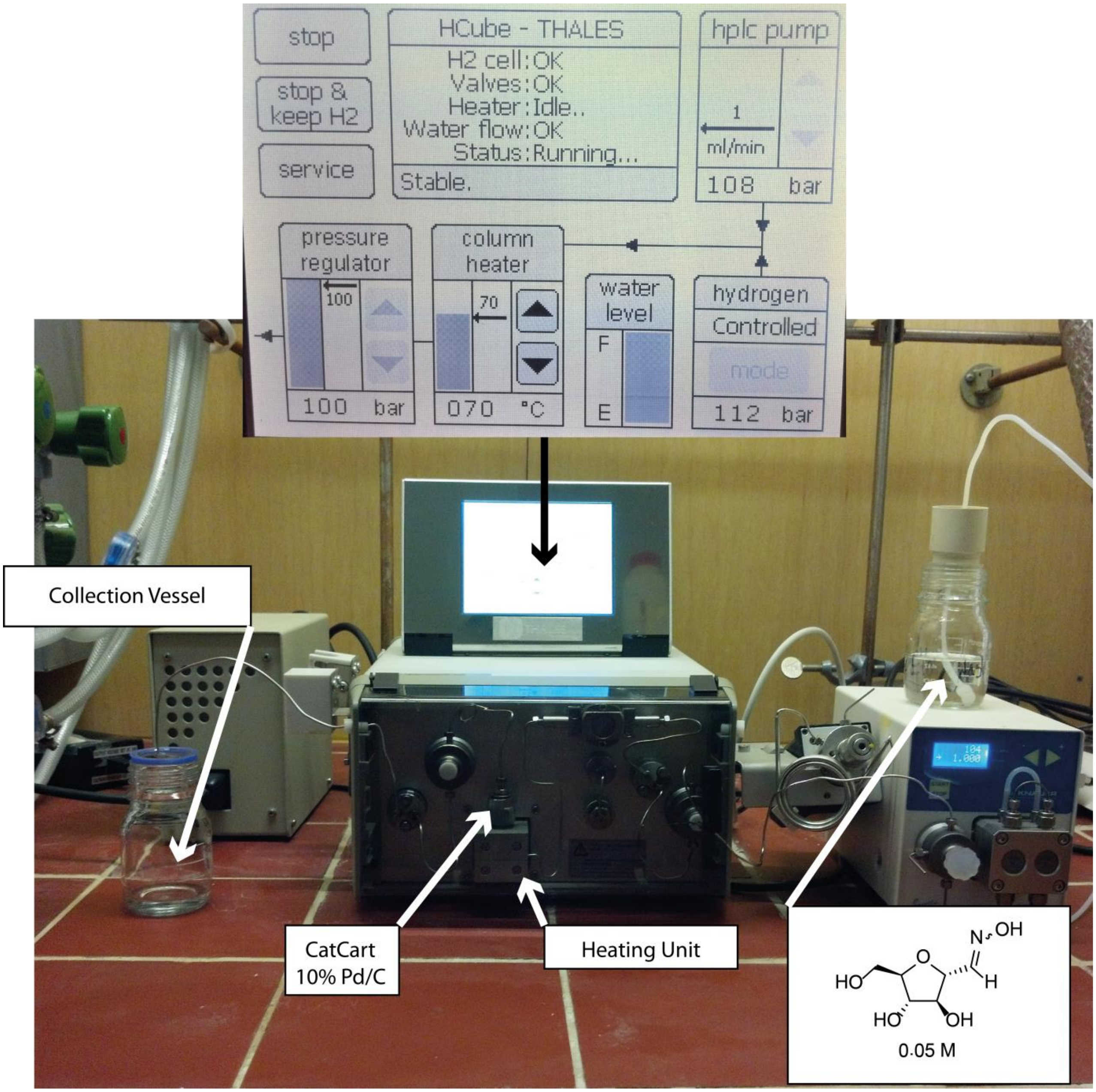

Figure S9: Experimental setup for of **3** using the Thales H-Cube.

5. NBDM

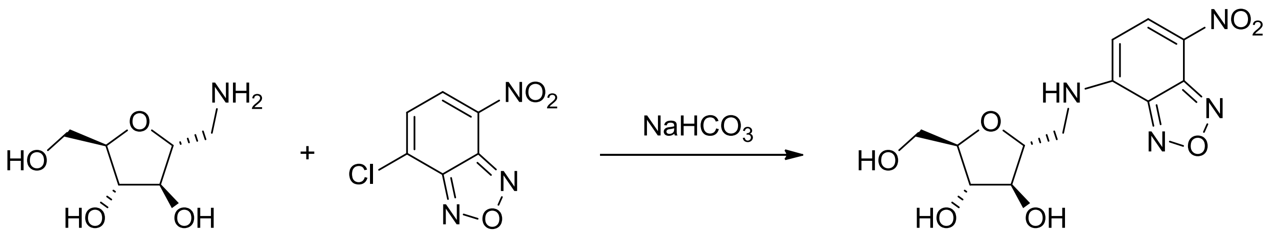

3

A 0.4 M solution of **3** in saturated sodium bicarbonate was reacted with equal volume of a 0.4 M solution of 4-chloro-7-nitrobenzofurazan in methanol using a syringe pump and heated tubing coil as shown in Figure S10. TLC of output sample indicated conversion to the product NBDM. ^{1}H NMR of crude reaction mixture indicates successful conversion to NBDM (Figure S11). The crude reaction mixture was concentrated in vacuo and then taken up in ethanol. The solids were filtered and the filtrate was concentrated onto silica gel before being loaded into a Biotage flash column with 30 g of dry silica gel. The column was equilibrated with pure ethyl acetate (5 CV) and then run at a gradient from 0–3% MeOH (30 CV). ^{1}H, ^{13}C, COSY and HSQC were consistent with literature.

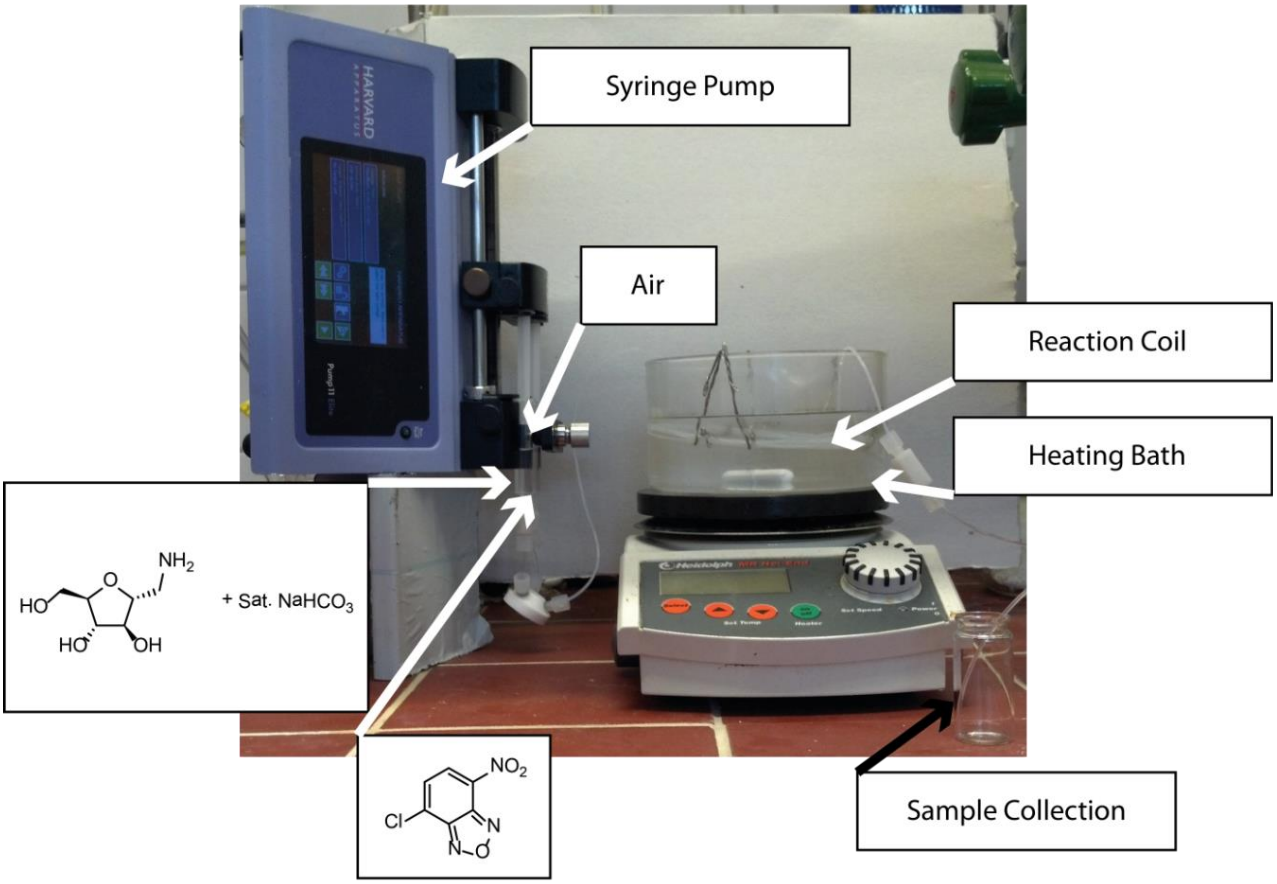

Figure S10: Set up for the conjugation NBD to **3**.

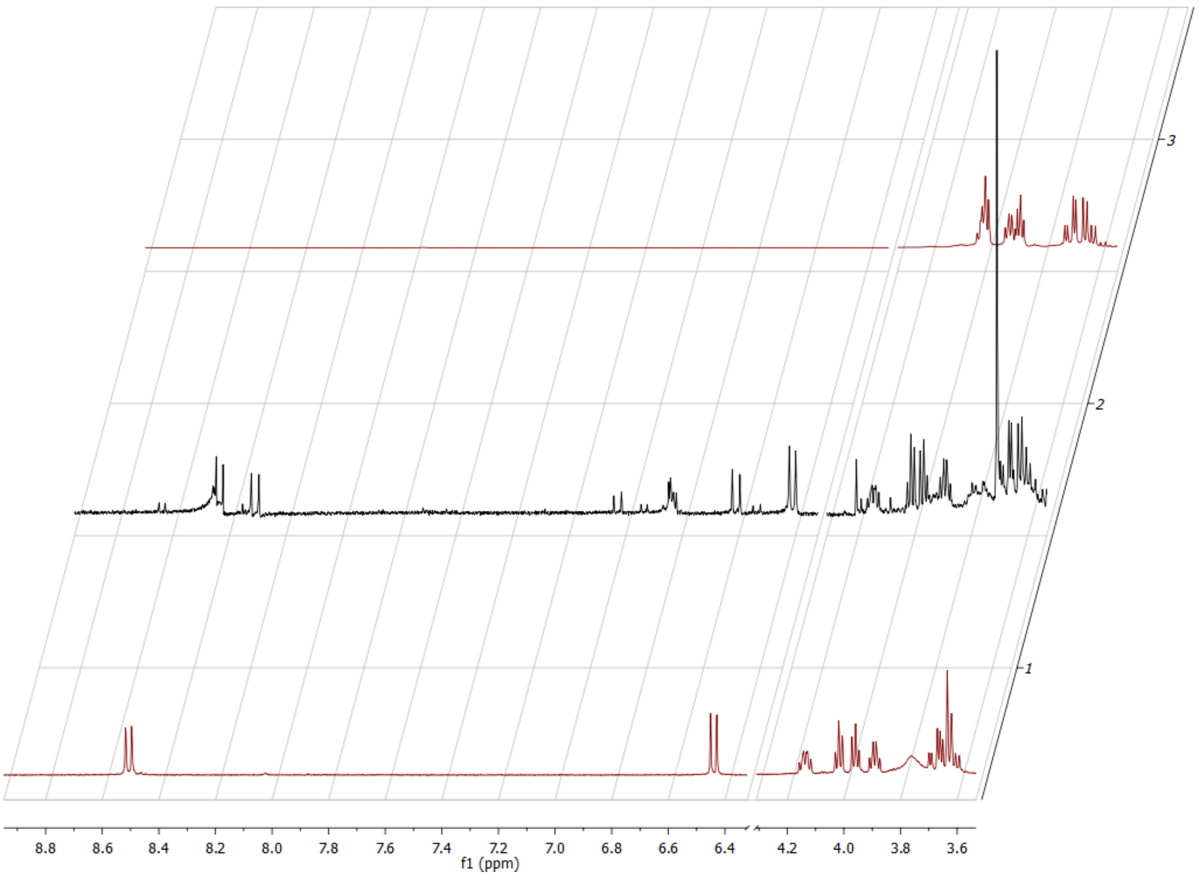

Figure S11: ^{1}H NMR of: top to bottom, **3**, Crude NBDM flow output, NBDM.

6. General fluorescence measurement procedure

Stock solutions of each of the possible quenchers and NBDM were prepared for making test solutions directly in a 96-well plate. The appropriate volume of quencher and NBDM stock solutions were added to the wells and then diluted with 1× PBS to a volume of 200 µL. Each fluorescence quenching experiment was done at a 2 µM NBDM. The wells were excited at 472 nm and fluorescence intensity was measured at 546 nm. For each well plate measured, eight wells containing only NBDM and eight wells with only quencher were used as positive and negative controls. Each concentration of NBDM or 2 µM NBDM with quencher was measured 3–6 times. For the quenching experiments the standard deviation and standard error of F_0/F was calculated using the following equations:

$$Y = \frac{F_0}{F}$$

$$s_Y = \sqrt{\left(\frac{\partial Y}{\partial F_0}\right)^2 {s_{F_0}}^2 + \left(\frac{\partial Y}{\partial F}\right)^2 {s_F}^2}$$

$$SE_Y = \frac{s_Y}{\sqrt{n}}$$

Figure S12: Equations used to calculate the standard deviation of F_0/F, s_y, and the standard error of F_0/F, SEy, where F_0 is the fluorescence intensity in the absence of a quencher and F is the fluorescence intensity in the presence of quencher.

Self-quenching data was fitted using the model for calcein self-quenching proposed by Memoli et al., *J. Pharm. Biomed. Anal.* **1999,** *19,* 627–632,

$$I = \beta_f\left(1 - 10^{-\varepsilon_m[C]}\right)e^{k_{\text{ife}}[C]}$$

Where I, represents the fluorescence intensity, β_f, is the proportionality constant, ε_m, is the molar extinction coefficient, k_{ife}, is the constant representing the inner filter effect, and [C] is the concentration of NBDM.

The quenching data for various fluorescence quenchers was fitted using a linear (dynamic or static quenching) or 2nd order polynomial fit (dynamic and static quenching).

$$\text{Dynamic:} \quad \frac{F_0}{F} = \frac{\tau_0}{\tau} = 1 + k_{SV}[Q]$$

$$\text{Static:} \quad \frac{F_0}{F} = 1 + k_a[Q]$$

$$\text{Dynamic + Static:} \quad \frac{F_0}{F} = (1 + k_{SV}[Q])(1 + K_a[Q])$$

or

$$\frac{F_0}{F} = 1 + (k_{SV} + k_a)[Q] + k_{SV}k_a[Q]^2$$

Dynamic and static quenching by themselves can be plotted using a linear fit in the form y = mx + b, where the fluorescence intensity in the absence of a quencher divided by the fluorescence intensity in the presence of a quencher, F_0/F, is directly proportional to the concentration of the quencher, [Q]. The Stern–Volmer constant, k_{sv}, or association constant, k_{a}, is represented by the slope of these plots. No lifetime measurements were made and therefore we cannot differentiate from dynamic and static quenching. When both dynamic and static quenching is occurring, F_0/F is related to the product of dynamic and static quenching, giving a 2nd order polynomial equation with respect to [Q], shown above.

7. NBDM self-quenching

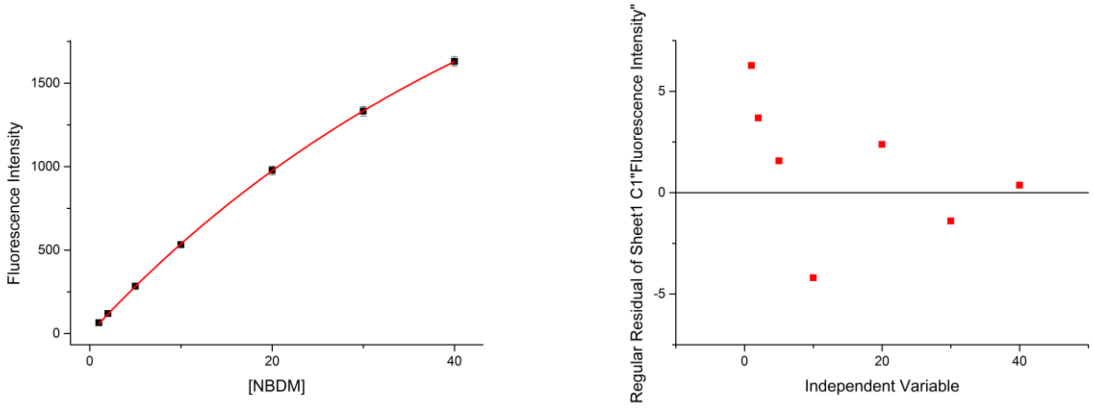

Figure S13: The fluorescence intensity of NBDM at a range of concentrations from 1-40 µM fitted using the calcein self-quenching model, $I = \beta_f\left(1 - 10^{-\varepsilon_m[C]}\right)e^{k_{\text{ife}}[C]}$ with direct weighting and residual plot.

Variables: $\beta_f = 2506.51155$, $\varepsilon_m = 10302.16812$, $k_{ife} = 1478.38419$

Residual sum of Squares: 519,98216

Adj. R-Square: 0,99997

8. Trypan Blue

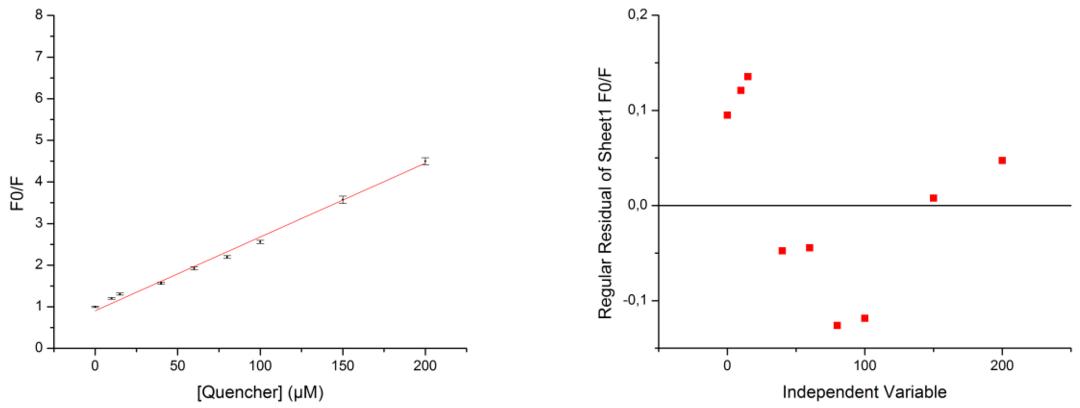

Figure S14: Stern–Volmer relationship (linear fit) and residual plot.

Residual sum of Squares: 0.00233

Adj. R-Square: 0.9951

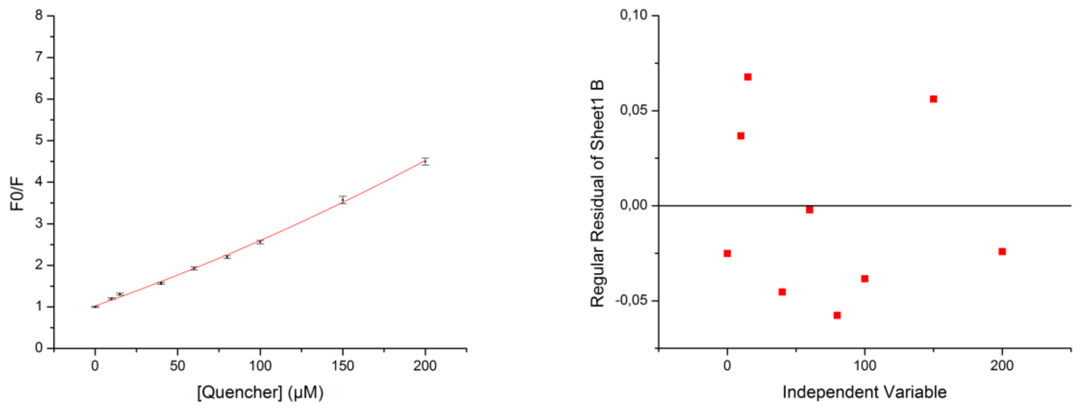

Figure S15: Static + Dynamic quenching model (polynomial fit, 2nd order) and residual plot. Equation

Variables: c = 1.02509, $(k_{SV} + k_a)$ = 0.01397, $k_{SV}k_a$ = 1.75986E−5

Residual sum of Squares: 7.01237E−4

Adj. R-Square: 0.99828

9. Bromophenol Blue

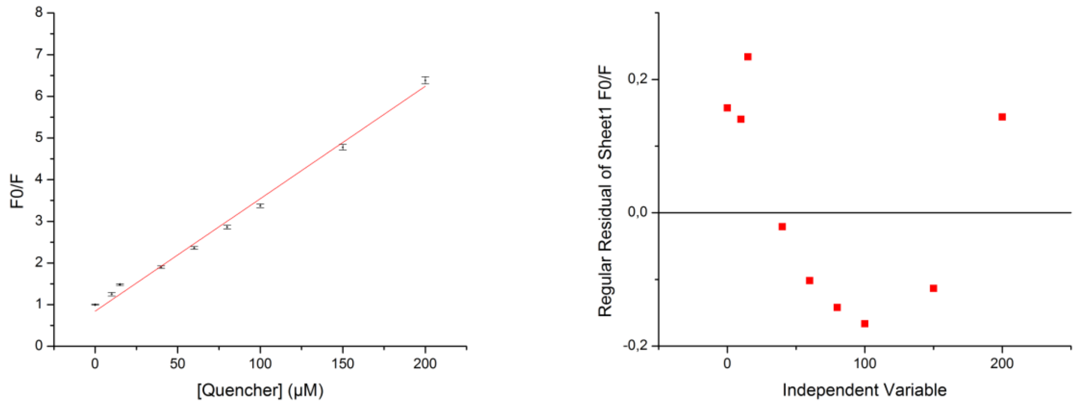

Figure S16: Stern–Volmer relationship (linear fit) and residual plot.

Residual sum of Squares: 0.00748

Adj. R-Square: 0.99364

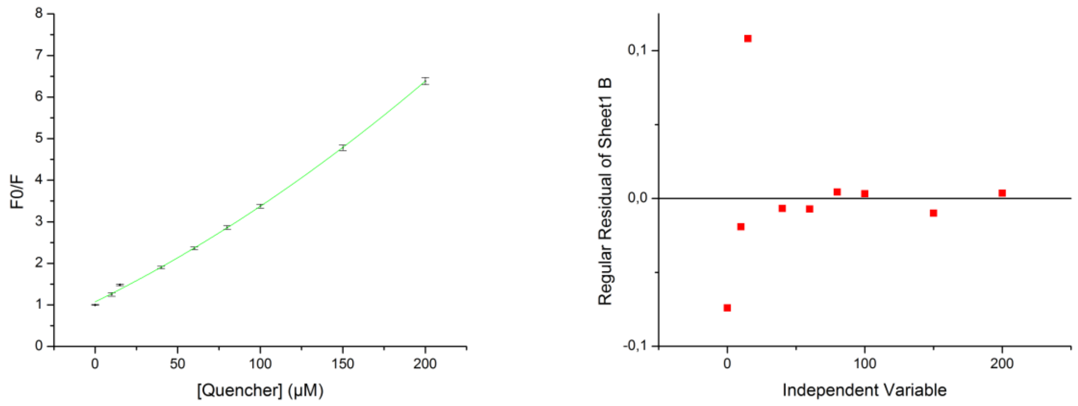

Figure S17: Static + Dynamic quenching model (polynomial fit, 2nd order) and residual plot.

Variables: c = 1.07157, $(k_{SV} + k_a)$ = 0.01948, $k_{SV}k_a$ = 3.53516E−5

Residual sum of Squares: 3.57233E−4

Adj. R-Square: 0.99965

10. Brilliant Blue R

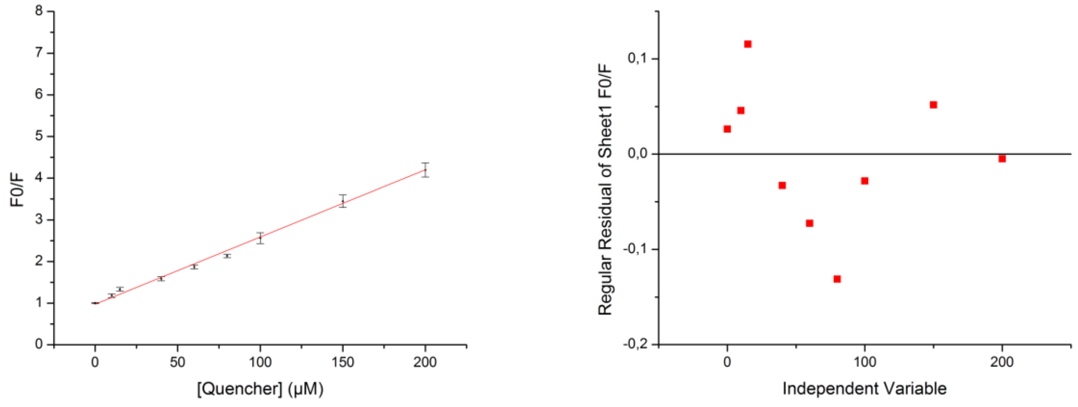

Figure S18: Stern–Volmer relationship (linear fit) and residual plot.

Residual sum of Squares: 0.00228

Adj. R-Square: 0.99672

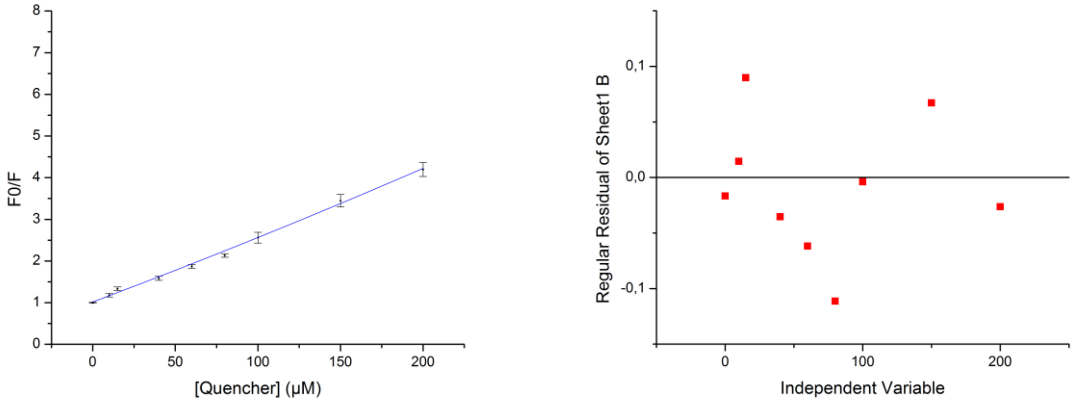

Figure S19: Static + Dynamic quenching model (polynomial fit, 2nd order) and residual plot.

Variables: c = 1.01659, $(k_{SV} + k_a)$ = 0.1491, $k_{SV}k_a$ = 5.6464E−6

Residual sum of squares: 0.00197

Adj. R-Square: 0.99669

11. Methylene Blue

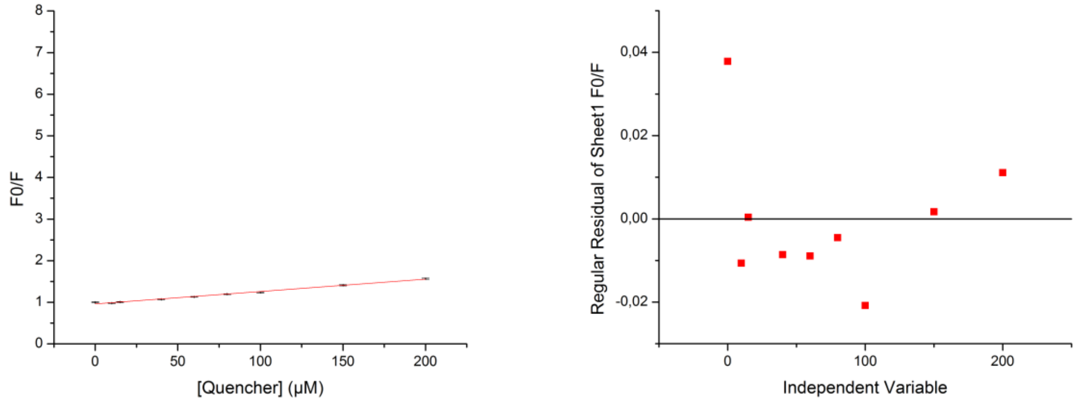

Figure S20: Stern–Volmer relationship (linear fit) and residual plot.

Residual sum of Squares: 2.43154E−5

Adj. R-Square: 0.99342

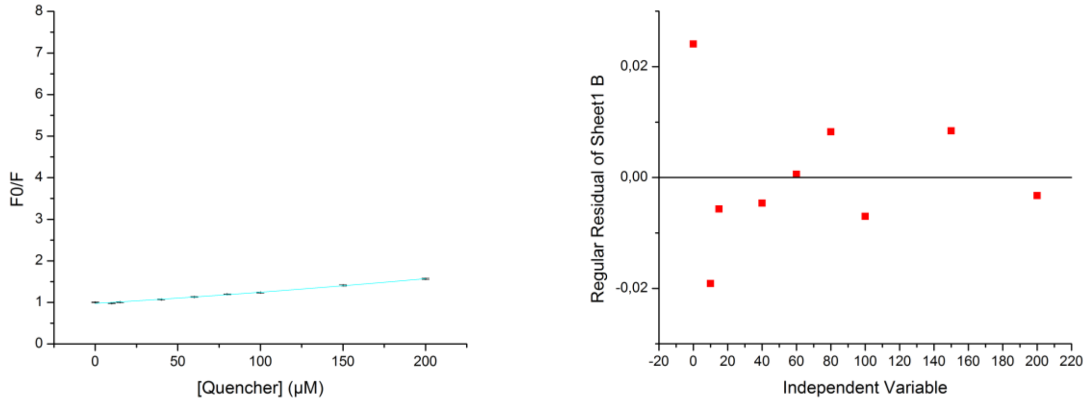

Figure S21: Static + Dynamic quenching model (polynomial fit, 2nd order) and residual plot.

Variables: c = 0.99608 $(k_{SV} + k_a)$ = .00242, $k_{SV}k_a$ = 2.78982E−6

Residual sum of squares: 1.24226E−5

Adj. R-Square: 0.99608

12. Methionine

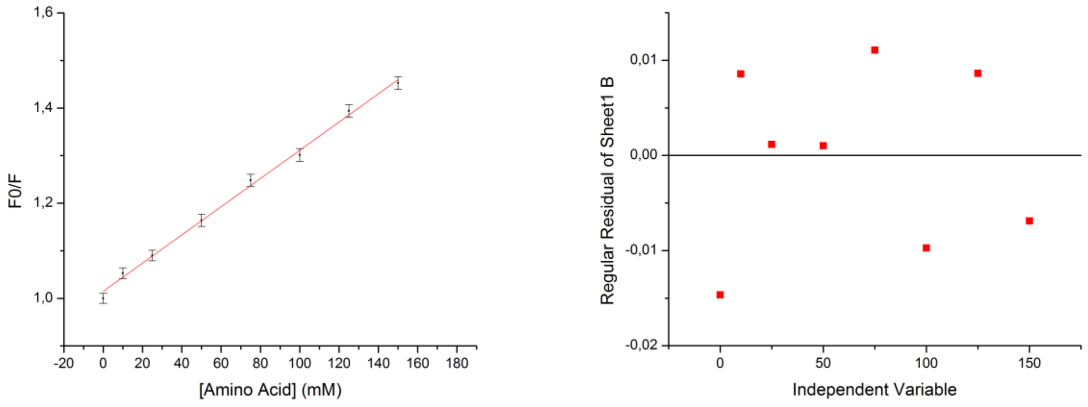

Figure S22: Stern–Volmer relationship (linear fit) and residual plot.

Variables: k_{SV} or k_a = 0.00297

Residual sum of Squares: 7.56755E−6

Adj. R-Square: 0.99614

13. Histidine

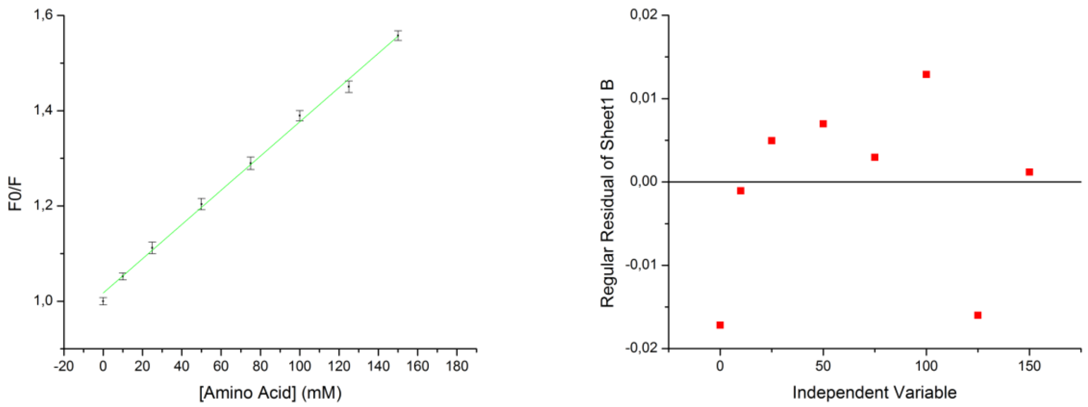

Figure S23: Stern–Volmer relationship (linear fit) and residual plot.

Variables: k_{SV} or k_a = 0.0036

Residual sum of Squares: 8.17753E−6

Adj. R-Square: 0.99642
